# Habitat Suitability of *Ziziphus spina‐christi* and *Ziziphus nummularia* in a Changing Climate in the Khalijo‐Omanian Zone, Iran

**DOI:** 10.1002/ece3.71406

**Published:** 2025-05-26

**Authors:** Saeed Behzadi, Gholamabbas Ghanbarian, Rasool Khosravi, Roja Safaeian, Hamid Reza Pourghasemi

**Affiliations:** ^1^ Department of Natural Resources and Environmental Engineering, School of Agriculture Shiraz University Shiraz Iran; ^2^ Department of Soil Science, School of Agriculture Shiraz University Shiraz Iran

**Keywords:** arid ecosystem, climate change impact, species distribution models, *Ziziphus* futures

## Abstract

Climate change, a global threat of utmost significance, has the potential to trigger shifts in biodiversity distribution and the emergence of novel ecological communities. While considerable research has focused on predicting the impacts of climate change on the range shift of species, a critical yet often overlooked aspect is the role of changing climate on plants in hot, arid, and poorly known ecosystems. We employed an ensemble species distribution modeling framework to investigate how climate change might affect the spatial range of two significant indicator species, *Ziziphus spina‐christi* and *Ziziphus nummularia* , within the hot and arid Khalijo‐Omanian ecosystem of Iran. We ran the models for the current species distribution using climatic variables and then projected the models for two future periods (2041–2070 and 2071–2100) under different climate scenarios. These findings suggest that both species respond differently to climate change under different climatic scenarios. Some regions may undergo range expansion, whereas others may experience range contraction due to shifting environmental conditions. Overall, both species are projected to shift their range towards higher latitudes as climatic conditions evolve. Conservation and management measures, including the identification of priority areas, are crucial for protecting these species. The conclusions of this study are valuable to biodiversity conservation authorities, local stakeholders, and individuals dedicated to preserving *Ziziphus* habitats.

## Graphical abstract

An ensemble species distribution modeling framework was used to investigate how climate change may affect the distribution of two *Ziziphus* species. Boyce index showed a strong predictive accuracy for *Z. spina‐christi* achieving (0.90), and for *Z. nummularia* (0.74), reflecting good predictive performance. Both species will respond variably to climate change under optimistic and pessimistic scenarios (2041–2070) and (2071–2100). Some regions may experience an expansion of their range, while others may see a contraction. Both species are projected to shift their potential habitat suitability toward higher latitudes as climate conditions evolve. Conservation and management measures, including identifying priority areas, are crucial to protect these species.

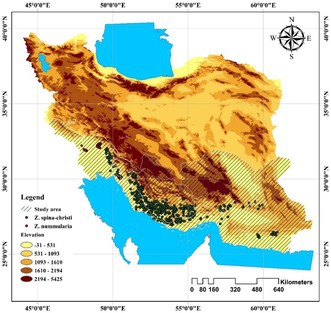

## 1 Introduction

The planet is experiencing significant transformations driven by a complex interplay of factors including rapid population growth, land use/land cover changes, and climate change. These shifts profoundly impact biological diversity, with consequences expected to become more severe as environmental conditions rapidly change. Consequently, conservation and environmental management are becoming increasingly challenging (Wiens et al. 2009).

The growing impact of anthropogenic climate change is evident in the alteration of global climate patterns, which directly affects species ranges. Projected increases in global mean temperatures, estimated to be between 0.3°C and 4.8°C by the end of the century (Stocker et al. 2014), are likely to drive latitudinal and altitudinal shifts in species range. Additionally, intensified extreme weather events such as floods and droughts can lead to widespread mortality and shift species range boundaries (Feng et al. 2024).

As anthropogenic climate change disrupts ecosystem structures and dynamics (Thuiller 2007; Weiskopf et al. 2020), species exhibit diverse responses including adaptation, shifts in phenology, and tracking suitable climatic conditions (Robinet and Roques 2010; Couet et al. 2022). Species unable to adapt to these changes may attempt migration; however, if they cannot disperse, they face the grim prospect of local or global extinction (Thuiller et al. 2008; Bellard et al. 2012). Climate change can induce shifts in species range at various scales, affecting both individual populations and ecosystems (Karl et al. 2009). Numerous studies have documented “uphill retreats” in various taxa as a response to climate change (Thomas et al. 2006).

Given the pervasive impact of ongoing climate change across the globe, recent research has focused on predicting its effects on species range. These efforts aim to develop management strategies to mitigate the adverse consequences (Huang et al. 2023; Chowdhury 2023; Song et al. 2023). Over the past two decades, ecologists have underscored the complexities of biodiversity forecasting and advocated for the adoption of predictive ecology (Mouquet et al. 2015). Nearly four decades of species distribution modeling (SDM) evolution have transitioned from simple snapshots of species distributions (Stanton et al. 2012) to essential tools for assessing species vulnerability in response to environmental changes (Guisan and Zimmermann 2000; Atwater and Barney 2021).

Understanding the impacts of climate change on species requires the integration of ecological niche concepts via SDMs (Wiens et al. 2009). SDMs are the most prevalent class of models in evolutionary and conservation ecology (Dormann et al. 2012), with applications that span crucial areas, such as endangered species protection, habitat management and restoration, and assessing risks posed by invasive species. By exploring the impacts of climate change on species ranges, researchers have enhanced predictive capabilities and developed strategies to mitigate adverse effects, highlighting the need for robust, ecologically informed management in the face of ongoing global climate change (Franklin 2010; Zurell et al. 2020). Numerous studies have investigated the repercussions of climate change on the potential spatial distribution of species (e.g., Behroozian et al. 2020; Baumbach et al. 2021; Karami et al. 2022; Naqinezhad et al. 2022; Wani et al. 2022; Shaban et al. 2023). The variability in predictions from different SDMs often presents challenges in the interpretation of results. To reduce the uncertainty in SDM forecasts, a practical approach is to employ an ensemble modeling framework that enhances the projection precision (Araujo and New 2007; Marmion et al. 2009; Naimi et al. 2022).

The genus *Ziziphus* sp. (Rhamnaceae) includes approximately 100–170 species of deciduous and evergreen trees and shrubs, which are primarily distributed in tropical and subtropical regions (Baghazadeh‐Daryaii et al. 2017). Among these species, Christ's thorn jujube (*Ziziphus spina‐christi* (L.) Desf.) is notable as a native and key species in the Middle East that exhibits remarkable resilience to drought and heat stress. The global range of this species encompasses North Africa, the Arabian Peninsula, India, Lebanon, Iraq, Pakistan, Afghanistan, and Iran (Saied et al. 2008; Rojas‐Sandoval 2017). In Iran, *Z. spina‐christi* is nearly ubiquitous in the Sahara‐Sindian (Khalijo‐Omanian) region. Another significant species, wild jujube ( *Z. nummularia* (Burm.f.) Wight & Arn.), is a deciduous shrub found in India, Pakistan, Iraq, and Iran (Pandey et al. 2010). Its distribution is primarily concentrated in Southwest Iran. Both *Z. spina‐christi* and *Z. nummularia* play vital roles in soil and water conservation, as well as in controlling wind and water erosion, thereby enhancing the overall resilience of ecosystems in arid and semi‐arid environments (Saied et al. 2008; Pandey et al. 2010; Rojas‐Sandoval 2017).

Although several studies have been conducted on the impact of climate change on the distribution patterns of some species within the genus *Ziziphus*, such as *Z. mauritania* and *Z. spinosa*, our understanding of the potential impact of climate change on these species remains limited (Zait and Schwartz 2018; Zhao et al. 2021; Muhammad et al. 2022). Predicting changes in species ranges under climate change is essential for effectively guiding conservation efforts. In this study, we employed SDMs to forecast the potential consequences of climate change on the range expansion or contraction of the two *Ziziphus* species. Our primary objectives were to explore the following questions: (1) how will the potential spatial distribution patterns of the two *Ziziphus* species transform in response to the challenges posed by climate warming? (2) What are the key climatic variables that significantly influence the distribution of these two species? The insights gained from this study will be instrumental in shaping effective conservation and management strategies for the preservation of these vital species.

## 2 Materials and Methods

### 2.1 Study Area

The study area encompasses the administrative borders of eight southern provinces of Iran, covering over 670,000 km^2^ the country's total area of 1.65 million km^2^. This region lies between latitudes 25°–40° N and longitudes 44°–64° E. Iran exhibits a diverse range of climatic conditions, with average annual temperatures varying from 20°C to 50°C and annual rainfall fluctuating between 100 mm and 2000 mm (Karimi et al. 2018; Farashi and Karimian 2021; Naderi Beni et al. 2021). One notable feature of Iran is its extraordinary variety of plant species, which is attributable to its unique geographical positioning at the intersection of three prominent phytogeographical regions: the Irano‐Turanian, Saharo‐Sindian, and Europe‐Siberian regions. This geographical convergence has transformed the Iranian Plateau into a cradle for evolutionary processes and a refuge for significant biodiversity, as well as serving as an essential ecological bridge that facilitates the exchange of flora and fauna between the eastern and western regions of Eurasia (Noroozi et al. 2019).

The Saharo‐Sindian phytogeographical region extends over a vast expanse, encompassing the western territories of India and Pakistan, the southern periphery of Iran, including the Persian Gulf and the Oman Sea, along with the Arabian Peninsula and Iraq, ultimately reaching North Africa. In Iran, this area is designated as the Khalijo‐Omanian zone, which comprises the eight provinces of Khuzestan, Kohgiluyeh and Boyer‐Ahmad, Fars, Bushehr, Hormozgan, Kerman, Ilam, and Sistan‐Baluchistan. It is important to highlight that the Khalijo‐Omanian arid ecosystem generally receives less than 100 mm of annual rainfall (Naderi Beni et al. 2021), with summers characterized by prolonged periods of extreme heat and drought (Sagheb Talebi et al. 2014).

### 2.2 Species Occurrence Localities

**FIGURE 1:**
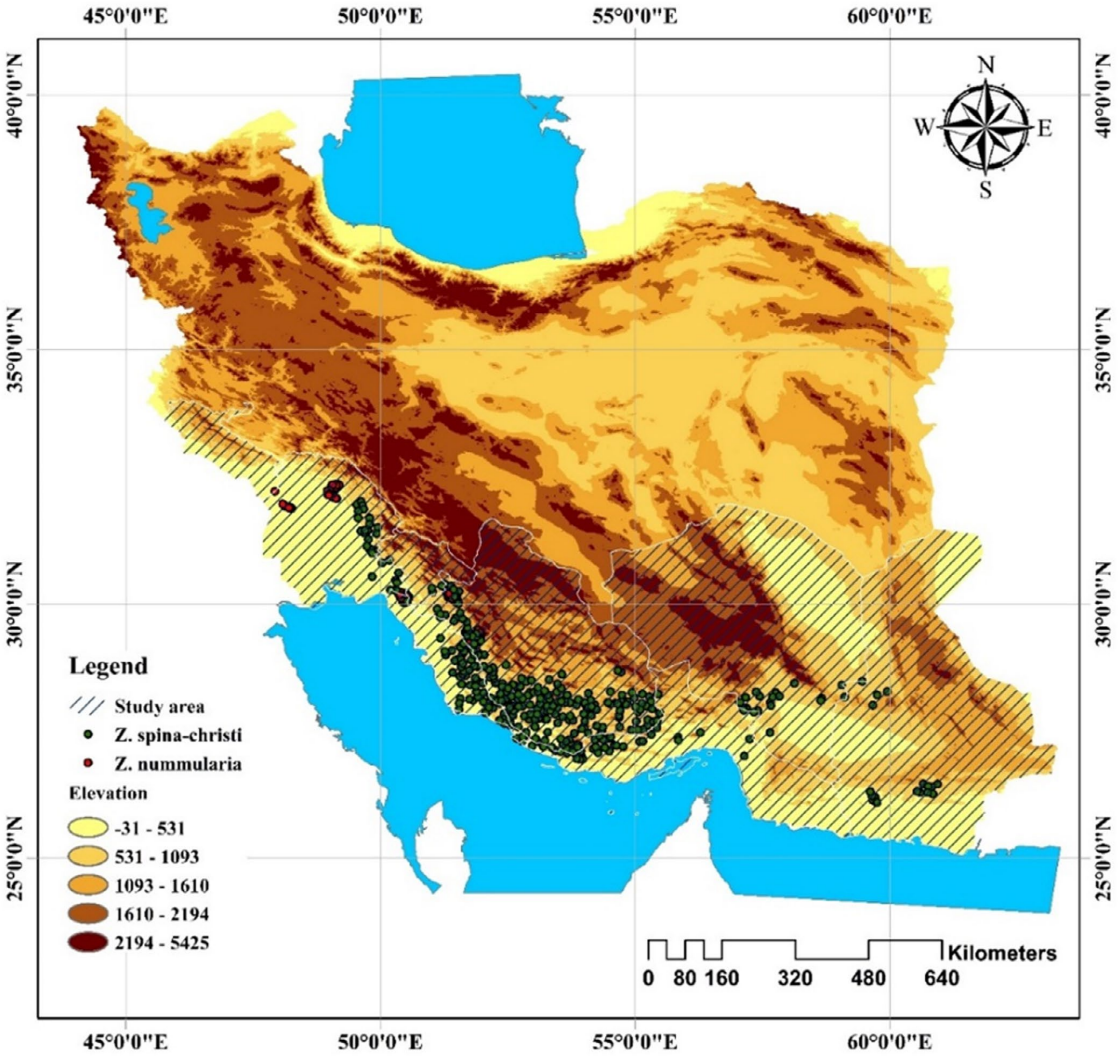
Map of the study area, including the presence localities of *Z. spina‐christi* and *Z. nummularia* .

The primary vegetation map for *Z. spina‐christi* and *Z. nummularia* was obtained from the Iran Research Institute of Forest and Rangeland (IRIFR), covering the period from 1988 to 2016. This map forms the basis for delineating the spatial distribution of these two species and identifying their respective occurrence locations. To obtain detailed information on the presence of *Z. spina‐christi* and *Z. nummularia* , field surveys were conducted between October 2021 and April 2022. Extensive field surveys reduce positional errors and ensure that presence data accurately represent suitable microhabitats. In addition, field surveys capture fine‐scale habitat variations, which are often missed in coarse distribution maps. We used distribution polygons solely to guide our fieldwork and identify potential areas of occurrence, ensuring comprehensive and unbiased sampling across the known range (Gábor et al. 2022). These efforts resulted in the identification of 419 and 161 locations in *Z. spina‐christi* and *Z. nummularia* . To mitigate spatial autocorrelation and prevent model overfitting, presence data were spatially filtered to a minimum of 1‐km distance from each other using the *SDMtoolbox* as described by Brown et al. (2017). Subsequently, a refined dataset consisting of 348 and 130 presence locations for *Z. spina‐christi* and *Z. nummularia* was used to construct the species distribution model (Figure 1).

### 2.3 Current and Future Climatic Variables

A dataset of 19 bioclimatic variables, with a spatial resolution of 30 arc‐seconds (approximately 1 km), was obtained from CHELSA version 2.1 (Karger et al. 2017) to map the current climatic niche of the species. We also obtained future climate data aligned with scenarios featured in the 6th Assessment Report of the Intergovernmental Panel on Climate Change (IPCC AR6) using the CHELSA version 2.1 database (http://chelsa‐climate.org), which provides a spatial resolution of 30 arc‐seconds (~1 km). This dataset includes two Shared Socioeconomic Pathway scenarios: SSP1‐2.6, which indicates an optimistic perspective, and SSP5‐8.5, which represents a pessimistic outlook. These climate projections were generated using the Global Circulation Model (GCM) GFDL‐ESM4 (Shaban et al. 2023; Mathias et al. 2023) and covered two time periods: 2041–2070 and 2071–2100 (Table S1).

To address collinearity issues among the variables, hierarchical cluster analysis was conducted using Pearson's correlation coefficient, with a cutoff threshold set at 0.7 (Gallego‐Narbón et al. 2023). This approach was executed using the R package *virtualspecies* v.1.6 (Leroy et al. 2016). To achieve this, we first identified groups of intercorrelated variables using a hierarchical ascending classification with a distance metric based on the Pearson's correlation coefficient. We then applied cluster analysis to the matrix of the predicted correlation coefficients to classify the variables into correlated groups. Subsequently, the models were run using each group of intercorrelated predictors, allowing for the selection of those with the highest contributions. The final model was calibrated using uncorrelated covariates that exhibited the highest percentage contributions (Louppe et al. 2020). In total, seven predictors were retained in the model using data from 2041 to 2070 and 2071 to 2100: mean diurnal range (bio2), isothermality (bio3), temperature seasonality (bio4), maximum temperature of the warmest month (bio5), precipitation of the wettest month (bio13), precipitation seasonality (bio15), and precipitation of the warmest quarter (bio18).

### 2.4 Climate Niche Modeling

We employed an ensemble modeling approach (Araujo and New 2007), using the average of three algorithms: generalized linear model (GLM; Nelder and Wedderburn 1972), generalized boosted model (GBM; Friedman 2001), and maximum entropy (MaxEnt; Phillips et al. 2006). To ensure consistency throughout the modeling process, we utilized the default parameters from the R package *biomod2* v.4.2–5‐2, as outlined by Thuiller et al. (2009), and ran each algorithm 10 times (Sadeghi et al. 2024). Owing to the lack of absence data in record‐based collections, we generated 10,000 pseudo‐absence locations randomly distributed throughout the study area (Sadeghi et al. 2024). To assess the predictive performance of the models, we divided the occurrence data into two subsets: 80% was designated for calibration, and the remaining 20% was used for evaluation.

We evaluated model performance using an independent test dataset with the Boyce index as the performance metric. The Boyce index is a better alternative for evaluating presence‐background models than other assessment metrics, as it quantifies the ratio of predicted presence to expected presence based on the habitat suitability generated by the models (Hirzel et al. 2006; Whitford et al. 2024). Its values range from −1 to +1, with positive values indicating a strong alignment with the observed distribution of presence, values close to zero reflecting performance akin to a random model, and negative values implying counterproductive predictions that indicate a low suitable area (Hirzel et al. 2006; Sillero et al. 2021). The Boyce index was calculated using the R package *ecospat* v.4.1.2 (Di Cola et al. 2017; Broennimann et al. 2022). We also computed the percentage contribution of each climatic variable to assess the impact of each variable on the potential distribution of species. For each algorithm, the measurement of each climate variable was divided by the total of all measurements for that specific algorithm and then multiplied by 100. This process is performed for all algorithms, and then the average is taken to show the percentage contribution of each variable relative to the others (Shaban et al. 2023).

Finally, we generated habitat suitability maps using ensemble species distribution modeling, categorizing the predictions into four distinct classes: (1) unsuitable, (2) low, (3) moderate, and (4) high suitability.

### 2.5 Climatic Niche Overlap and Similarity

To compare the climatic niches of *Z. spina‐christi* and *Z. nummularia* and gain further insights into the patterns of co‐occurrence, we conducted niche overlap and similarity analyses in *ecospat*. We calculated Schoener's D index to assess species niche overlap. This metric ranges from 0 to 1, where 0 indicates no overlap between species and 1 indicates complete overlap. Niche similarity was tested to compare the ecological niches of the two species, given habitat availability. The similarity test compares the observed niche overlap values to the null distribution of niche overlap calculated from the environmental background occupied by the two species (Broennimann et al. 2012). Significantly higher overlap values indicated higher niche similarity than expected from the null distribution, whereas significantly lower overlap values may indicate niche divergence. As the distribution of *Z. nummularia* occurrence localities was restricted mainly to the southwest of Iran, while *Z. spina‐christi* had records throughout the south and southwest of the country, we generated minimum convex polygons (MCPs) based on the occurrence points of the two species to define the geographic range of each species. This range was used to extract the PC scores from all grid cells masked by the MCP to define the environmental background. We began by performing principal component analysis (PCA) of environmental variables across the background area using the R package *ade4* v.1–22 (Dray and Dufour 2007). This analysis incorporated 19 bioclimatic variables, with the first two principal component axes used to define two‐dimensional environmental space. The first two PCA axes were used to estimate the kernel densities. The PC scores were extracted from the occurrence points to define the environmental space occupied by each species. The environmental space was divided into a grid of 100 × 1000 cells to produce an occurrence density grid for each species. We subsequently performed 100 replicates of the niche similarity test to determine the statistical significance. To provide a more comprehensive understanding of climate niche dynamics, three additional indicators were computed: “niche stability,” “niche expansion,” and “niche unfilling” (Datta et al. 2019; Bates and Bertelsmeier 2021).

## 3 Results

### 3.1 Climate Niche Modeling

The models exhibited remarkable predictive powers for both species. Notably, the evaluation of the ensemble species distribution model revealed a Boyce index of 0.90 for the model of *Z. spina‐christi*, indicating high predictive accuracy. In contrast, the *Z. nummularia* model exhibited a Boyce index of 0.74, reflecting a good level of predictive accuracy.

For *Z. spina‐christi*, the ensemble species distribution model identified the main factors influencing its climatic niche as the maximum temperature of the warmest month (42.24%), precipitation of the wettest month (24.88%), and precipitation of the warmest quarter (10.11%). In contrast, *Z. nummularia's* distribution was primarily influenced by precipitation during the wettest month (34.65%), isothermality (18.29%), and the maximum temperature of the warmest month (13.92%).

**FIGURE 2:**
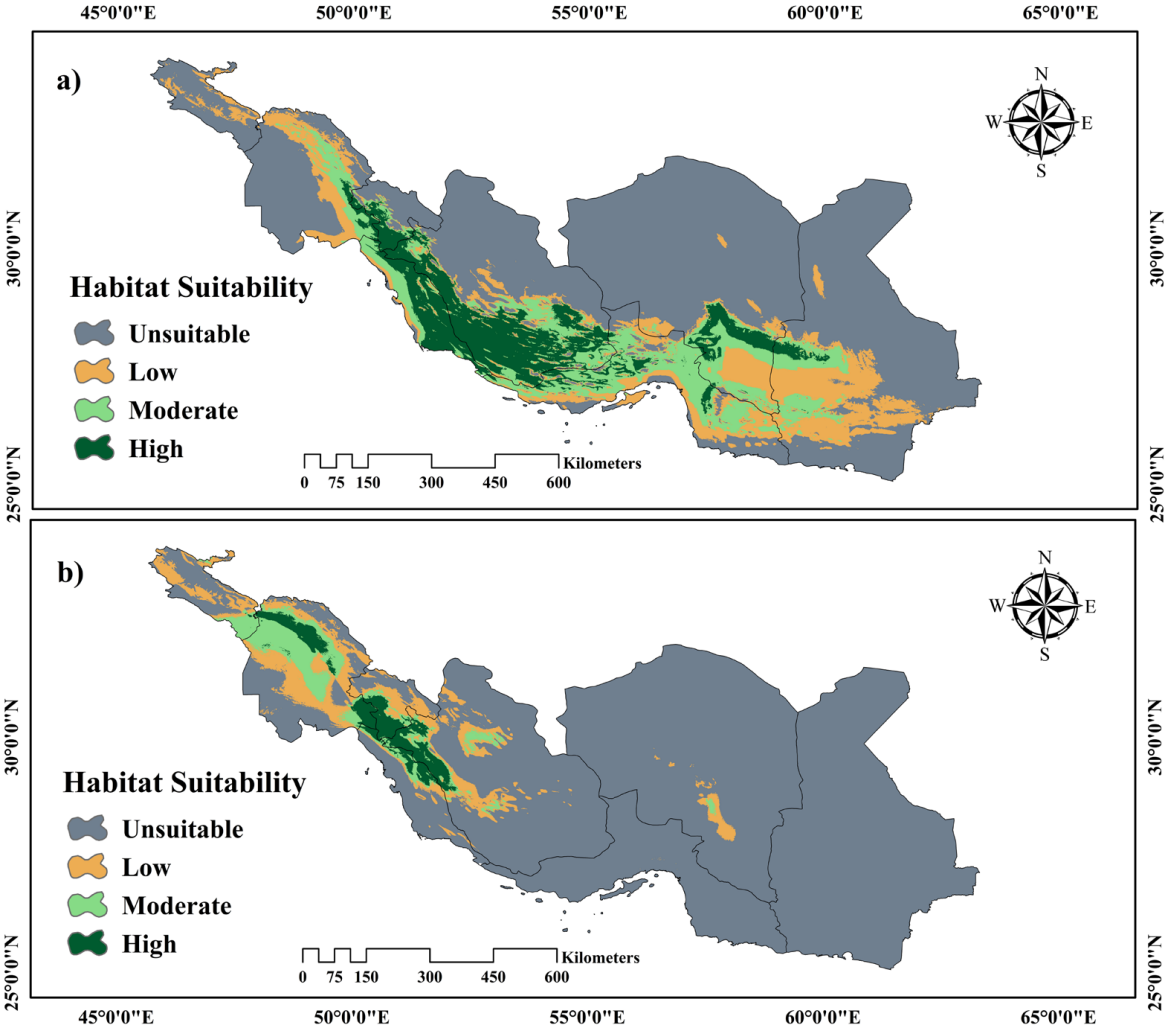
Habitat suitability map under current climate conditions based on the ensemble species distribution model for (a) *Z. spina‐christi and* (b) *Z. nummularia* .

The habitat suitability map, created using the ensemble species distribution modeling framework, reveals that under current climatic conditions, approximately 72,544.05 km^2^ (10.69%) of the study area encompassing 670,000 km^2^ are expected to be highly suitable for *Z. spina‐christi*. This area primarily includes the southern regions of Fars, Bushehr, parts of Kerman, and Kohgiluyeh and Boyer‐Ahmad provinces, situated within the Saharo‐Sindian phytogeographical region. In contrast, the potential distribution of *Z. nummularia* is more limited, with around 20,325.36 km^2^ (2.99%) of the study area projected as highly suitable under current climatic conditions. This distribution is concentrated in the southwestern regions of Fars, Kohgiluyeh, Boyer‐Ahmad, and parts of the Khuzestan province (Figure 2).

**FIGURE 3:**
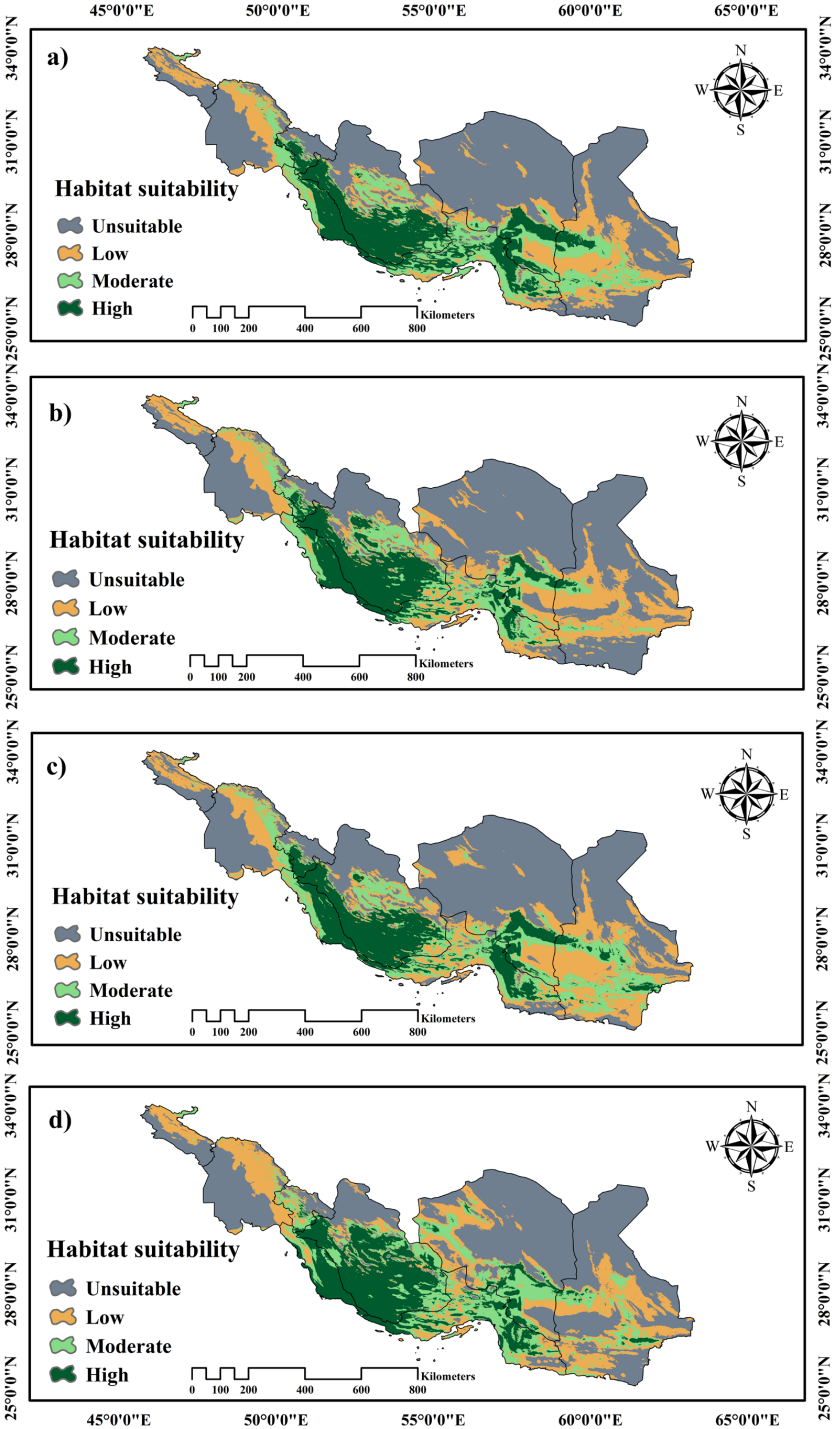
Mapping suitable habitats for *Z. spina‐christi* under climate change scenarios: (a) SSP1‐2.6 (2041–2070); (b) SSP5‐8.5 (2041–2070); (c) SSP1‐2.6 (2071–2100); (d) SSP5‐8.5 (2071–2100).

It is clear that each species experienced a gradual transition across different time periods and habitats in response to climate change, as illustrated in Figure 3. For the future potential distribution of *Z. spina‐christi*, a decline in unsuitable habitat class is predicted across both climate scenarios (SSPs) and time periods (2041–2070 and 2071–2100).

In regions with suitable potential, some areas may experience both increases and decreases in habitat range. However, the overall trend indicates growing potential for highly suitable habitats. Future climate predictions suggest that suitable habitats may emerge in Hormozgan Province, particularly in areas currently dominated by various species of *Acacia* (Sagheb Talebi et al. 2014; Zarei et al. 2015).

Conversely, the SSP5‐8.5 scenario indicates that the projected rise in global temperatures from 2071 to 2100 is expected to have a considerable effect on the habitat of *Z. spina‐christi*. This could make vital habitats in the Kohgiluyeh and Boyer‐Ahmad provinces unsuitable, leading to a decrease in the potential of areas now considered suitable. Furthermore, a shift towards higher latitudes is forecasted, resulting in the fragmentation of potential habitats in Kerman and Hormozgan provinces and a reduction in spatial integrity (as illustrated in Figure 3 and detailed in Tables 1 and 2).

**TABLE 1:** Habitat suitability class areas for *Z. spina‐christi* under climate change scenarios.

SSP	Time periods	Unsuitable km^2^/%	Low km^2^/%	Moderate km^2^/%	High km^2^/%
	1981–2010	421,485.06 62.14%	101,214.18 14.92%	83,020.73 12.24%	72,544.04 10.69%
SSP1‐2.6	2041–2070	336,683.07 49.63%	124,449.10 18.34%	104,880.98 15.46%	112,250.86 16.54%
	2071–2100	318,098.15 46.89%	153,301.37 22.60%	102,505.72 15.11%	104,358.77 15.38%
SSP5‐8.5	2041–2070	345,713.29 50.97%	150,677.47 22.21%	83,765.88 12.35%	98,107.37 14.46%
	2071–2100	304,067.27 44.83%	148,373.25 21.87%	108,054.29 15.93%	117,769.20 17.36%

**TABLE 2:** Range changes suitable habitat for *Z. spina‐christi*.

SSP	Time periods	Unsuitable %	Low %	Moderate %	High %
SSP1‐2.6	2041–2070	−12.51	+3.42	+3.22	+5.85
SSP5‐8.5	2041–2070	−11.17	+7.29	+0.11	+3.77
SSP1‐2.6	2071–2100	−15.25	+7.68	+2.87	+4.69
SSP5‐8.5	2071–2100	−17.31	+6.95	+3.69	+6.67

**FIGURE 4:**
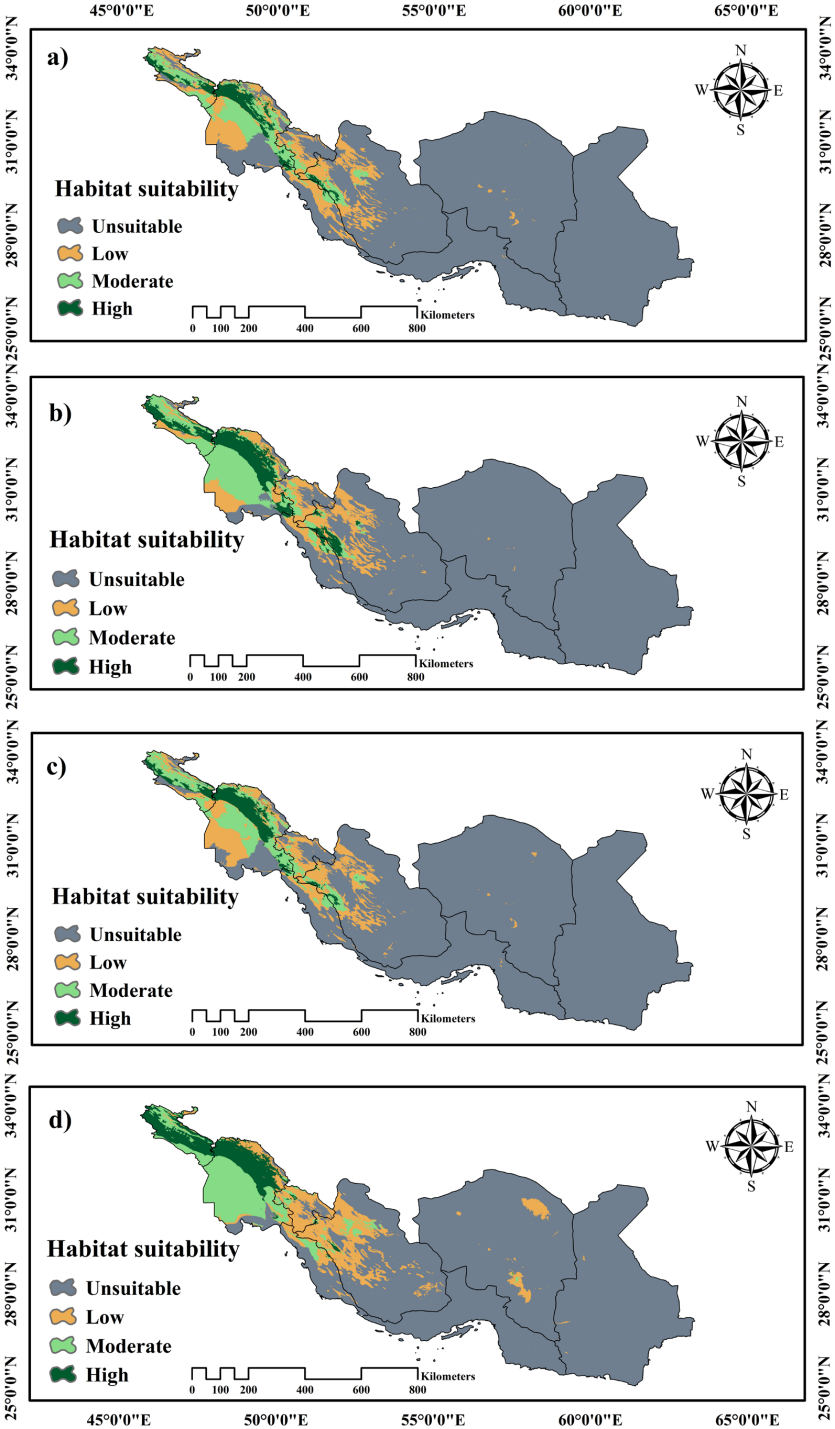
Mapping suitable habitats for *Z. nummularia* under climate change scenarios: (a) SSP1‐2.6 (2041–2070); (b) SSP5‐8.5 (2041–2070); (c) SSP1‐2.6 (2071–2100); (d) SSP5‐8.5 (2071–2100).

Predicted climate change is expected to enhance the suitability of potential habitats for *Z. nummularia* at higher latitudes. However, emerging forecasts indicate that the habitats currently deemed suitable in the Fars, Kohgiluyeh, and Boyer‐Ahmad provinces are likely to become unsuitable as the climate continues to evolve. Conversely, the potential habitat for *Z. nummularia* is projected to thrive in Khuzestan Province, with a new suitable habitat expected to arise in Ilam Province. These transformations highlight the fluid nature of habitat suitability in response to climate change (Figure 4 and Tables 3 and 4).

**TABLE 3:** Habitat suitability class areas for *Z. nummularia* under climate change scenarios.

SSP	Time periods	Unsuitable km^2^/%	Low km^2^/%	Moderate km^2^/%	High km^2^/%
	1981–2010	579,825.64 85.48%	49,770.12 7.33%	28,342.90 4.17%	20,325.36 2.99%
SSP1‐2.6	2041–2070	569,516.73 83.96%	58,937.12 8.68%	34,210.39 5.04%	15,599.78 2.29%
	2071–2100	567,817.85 83.71%	56,446.99 8.32%	36,368.75 5.36%	17,630.42 2.59%
SSP5‐8.5	2041–2070	555,075.48 81.83%	49,975.67 7.36%	45,538.78 6.71%	27,674.08 4.08%
	2071–2100	534,805.29 78.84%	61,661.53 9.09%	50,729.14 7.47%	31,068.06 4.58%

**TABLE 4:** Range changes suitable habitat for *Z. nummularia* .

SSP	Time periods	Unsuitable %	Low %	Moderate %	High %
SSP1‐2.6	2041–2070	−1.52	+1.35	+0.87	−0.70
SSP5‐8.5	2041–2070	−3.65	+0.03	+2.54	+1.09
SSP1‐2.6	2071–2100	−1.77	+0.99	+1.19	−0.40
SSP5‐8.5	2071–2100	−6.64	+1.76	+3.30	+1.59

### 3.2 Climate Niche Divergence

**FIGURE 5:**
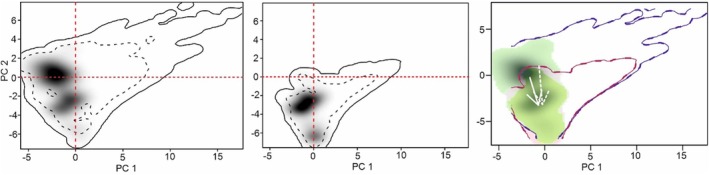
A visualization of realized niche dynamics, comparing the two *Ziziphus* species within the climatic space based on Principal Component Analysis (PCA‐env). In this two‐dimensional plot, colors signify niche characteristics, with green indicating stability, gray representing unfilling, and yellow signifying expansion. White arrows denote the shift in the center of the species' niche (solid) and describe the background conditions (dashed). The darker shading indicates the density of species occurrences. The solid line delineates the full range (100%) of climatic niche breadths for pairs of compared species. Conversely, the dashed line represents 50% of the environmental density, marking the boundary climate conditions for defining the niches of *Z. spina‐christi* (blue) and *Z. nummularia* (red).

The initial two PCA axes accounted for 54.12% and 26.00% of the total variance, respectively. There was a considerable niche overlap between the two species (Schoener's SD = 0.47). The niche similarity results showed that the observed niche overlap did not differ from random expectations (*p* > 0.01). When calculating climatic niche dynamic indices, which indicate niche shifts under similar conditions, it was evident that both species showed large niche stability (98.65%), low niche unfilling (16.80%), and niche expansion (1.34%) (Figure 5).

## 4 Discussion

As human activities continue to modify climatic conditions, the future viability and distribution of numerous species face significant threats (Gaur and Squires 2018).

To effectively address the challenges of biodiversity conservation, it is crucial to predict the potential habitat ranges of species and map their potential spatial distributions under projected climate scenarios. This task is especially vital for the Saharo‐Sindian ecosystem because of its limited resilience to environmental changes (Vaghefi et al. 2019; Abo Hatab et al. 2024).

Our findings indicated that both species are projected to experience significant shifts in suitable habitats under future climate scenarios. Notably, the distribution of *Z. spina‐christi* is predicted to contract in lowland arid regions, while expanding to higher elevations where cooler conditions may become more favorable (Thomas et al. 2004; Parmesan 2006). In contrast, *Z. nummularia* is likely to experience a substantial reduction in its range, particularly in areas with increasing temperatures and decreasing precipitation (IPCC 2021). These findings underscore the need to prioritize conservation efforts in regions forecasted to remain climatically suitable and monitor areas at risk of habitat loss.

The identification of potential refugia is essential to support the long‐term persistence of these species. Our models identify specific regions projected to remain climatically stable under various scenarios, thereby representing priority areas for in situ conservation efforts (Hannah et al. 2002). For *Z. spina‐christi*, conservation initiatives should focus on protecting emerging suitable zones at higher altitudes. Meanwhile, *Z. nummularia* requires targeted efforts to maintain its current population in regions predicted to experience the most significant habitat contraction. Establishing protected areas and ecological corridors between fragmented populations can facilitate species dispersal and reduce the risk of local extinction (Krosby et al. 2010).

In addition, these findings have practical implications for habitat restoration and species management. For instance, areas identified as future refugia can serve as priority zones for reforestation or assisted migration programs (Hoegh‐Guldberg et al. 2008). Tailored conservation strategies that account for species‐specific vulnerability and habitat dynamics are essential. Furthermore, ex situ conservation methods such as seed banking or establishing living collections should be considered to preserve genetic diversity and provide resources for future restoration efforts (Hoban et al. 2020).

Beyond species‐specific conservation, the preservation of *Z. spina‐christi* and *Z. nummularia* is vital for maintaining ecosystem function and supporting local communities. Both species play an essential role in providing food and shelter for wildlife and offer socio‐economic benefits through their use in traditional medicine, food for livestock, and agroforestry (Shackleton et al. 2007). The loss of these species from vulnerable regions could disrupt ecological interactions and threaten the livelihoods of the communities reliant on them. Consequently, incorporating our species distribution models into regional land‐use planning and conservation policy is vital for the protection of biodiversity and the maintenance of the ecosystem services these species support (Pereira et al. 2010; Lu et al. 2024).

Our research identified the maximum temperature of the warmest month (bio5) as the most significant predictor of *Z. spina‐christi* distribution. This aligns with the known preference of the species for high‐temperature environments. *Z. spina‐christi* is recognized as a thermophilic tree that is highly tolerant to heat and drought and thrives in regions with mean annual temperatures between 19°C and 28°C. It is frost‐sensitive and predominantly found in desert and arid areas, indicating that its distribution is limited by lower temperatures (Rojas‐Sandoval 2017). Studies have shown that while germination can occur at moderate cold stress (22/16°C), seedling growth and photosynthetic capacity are significantly reduced, highlighting temperature as a limiting factor for successful establishment (Zait et al. 2020).

In contrast, for *Z. nummularia* , the key determinant was precipitation during the wettest month. This is consistent with the adaptability of the species to various ecological habitats, including hills, ravines, plains, and cultivated fields. *Z. nummularia* thrives in areas with mean annual rainfall ranging from 100 to 1000 mm and can grow on very shallow and skeletal soils, gravelly plains, sand dunes, alluvium, and rocky areas (Mesmar et al. 2022).

Its ability to thrive in extreme habitats, particularly arid regions, underscores the importance of precipitation patterns in determining its distribution. The importance of moisture availability during critical growth periods is a common theme in plant distribution studies (Ghrabi‐Gammar et al. 2021; Singh and Meghwal 2020; Kaur et al. 2023). This precipitation dependency is not limited to *Ziziphus* but appears to be a common trait among plant species adapted to seasonally dry environments. Studies on *Senegalia senegal*, another dryland‐adapted species, have identified precipitation of the wettest month as one of the primary environmental determinants of its distribution, along with seasonal potential evapotranspiration, soil pH, and mean temperature of the wettest quarter (Lyam et al. 2020). Similarly, *Sambucus williamsii* demonstrated a pronounced reliance on precipitation during the wettest month as a key factor determining its distribution range (Luo et al. 2023).

Future research on *Z. nummularia* could incorporate a wider range of environmental variables to provide a more comprehensive understanding of its distribution limits.

Interestingly, our findings diverge from those of Ksiksi et al. (2019), who identified three main predictors influencing the geographical distribution of *Z. spina‐christi* in the United Arab Emirates. Their study emphasized the importance of precipitation during the coldest quarter, annual precipitation, and the mean diurnal range, which collectively represented approximately 80% of the total variable contribution for this species. This discrepancy underscores the complexity of the ecological factors involved and encourages further exploration of the environmental influences shaping these species' distributions. One potential explanation for these differences is that different environmental variables may act as limiting factors across various parts of the species range. Species distributions are rarely governed by a single environmental driver. Instead, they result from the combined influence of multiple interacting ecological processes. As environmental conditions vary spatially, the relative importance of different variables can shift across a species' geographical range (Austin and Van Niel 2011).

In some regions, climatic factors, such as temperature and precipitation, may play a dominant role in limiting the distribution of *Z. spina‐christi*, whereas in other areas, soil properties, competition, or other biotic interactions may be more influential. This spatial heterogeneity in limiting factors can lead to varying predictions depending on the environmental context and specific climate scenarios examined. Such variations are particularly pronounced in species with broad ecological amplitudes, as they can tolerate a range of conditions but may exhibit differential sensitivity to particular factors in different parts of their distribution (Guisan and Thuiller 2005).

Moreover, the interaction between biotic and abiotic factors can further complicate distribution modeling outcomes. For instance, biotic interactions, such as competition, herbivory, or mutualism, can mediate the impact of environmental variables on species distributions (Sexton et al. 2009). Failure to account for these dynamic relationships may contribute to discrepancies between the model predictions and observed patterns.

Future research could benefit from employing fine‐scale environmental data and incorporating biotic interactions to refine the predictions. Additionally, evaluating how these factors change under future climate scenarios will provide a more comprehensive understanding of the potential distributional shifts of species and the underlying causes of the observed discrepancies. (Guisan and Thuiller 2005; Sexton et al. 2009; Austin and Van Niel 2011).

### 4.1 Range Shift of *Z. spina‐christi* and *Z. nummularia*

The current potential distribution of *Z. spina‐christi* is mainly projected for the southern regions of Fars, parts of Bushehr, areas south of Kohgiluyeh and Boyer‐Ahmad, and Kerman provinces. The presence of climatically suitable habitats is notably limited in Khuzestan and the southeastern regions, especially in Hormozgan and Sistan‐Baluchistan. This area is situated within the Khalijo‐Omanian zone, a subregion of the Saharo‐Sindian phytogeographical region in Iran, and is further divided into two distinct territories: Khaliji and Omani. Each territory has unique ecological characteristics and varying climatic conditions, with the coastal areas of Omani experiencing higher temperatures. Notably, the average temperature across this zone exhibits an increasing gradient from west to east (Sagheb Talebi et al. 2014).

Within Khaliji territory, *Z. spina‐christi* plays a crucial ecological role. In contrast, the Omani territory is dominated by plant communities primarily composed of the *Acacia* genus, which is essential to the region's woody flora (Sagheb Talebi et al. 2014). The observed decline in habitat suitability for *Z. spina‐christi* in southeastern Iran reflects the ongoing transformations in plant communities. Our study revealed significant differences between the two ecological zones. In an optimistic scenario (SSP1‐2.6), projections for the periods 2041–2071 and 2071–2100 indicate a potential expansion of *Z. spina‐christi's* distribution in parts of Fars, Kerman, and Bushehr. This scenario suggests the emergence of suitable habitats in the eastern region and the development of such habitats in western Hormozgan. Conversely, the SSP5‐8.5 scenario for the period 2041–2070 predicts a contraction and reduced viability of potential habitats in Kohgiluyeh and Boyer‐Ahmad. Additionally, suitable habitats in southern Kerman and Hormozgan are expected to become fragmented, while habitat expansion in Fars is forecasted to shift towards higher latitudes. As projected under the SSP5‐8.5, rising temperatures are expected to cause a significant decrease in suitable habitats for *Z. spina‐christi* in southern Kerman between 2071 and 2100. Simultaneously, the Fars province is expected to experience the relocation of suitable habitats towards higher latitudes. Additionally, Kohgiluyeh and Boyer‐Ahmad provinces, currently considered potentially favorable habitats for this species, may become increasingly unsuitable due to forthcoming climate changes.

Despite the existing challenges, the overall trend indicates an expected increase in habitat suitability for *Z. spina‐christi*. This finding aligns with various global studies that have recorded similar shifts in species range (Hänke et al. 2016; Ksiksi et al. 2019; Li et al. 2019; Zhao et al. 2021; Al‐Munqedhi et al. 2022; Xiao et al. 2023; Idi et al. 2024). These studies emphasize the complex and often unpredictable nature of species' responses to climate change.

The current potential distribution of *Z. nummularia* is primarily concentrated in the western regions of Fars Province, northern Bushehr Province, southern Kohgiluyeh and Boyer‐Ahmad, and parts of Khuzestan, which are highly suitable for this species. Typically, this species is found as a companion within *Z. spina‐christi* vegetation communities, but it does not form distinct plant communities (Sagheb Talebi et al. 2014) While the available information is limited, it suggests that both *Z. spina‐christi* and *Z. nummularia* are well adapted to arid and semi‐arid environments, with a high tolerance for water scarcity and potentially high temperatures. More specific research on the environmental demands of these species, particularly *Z. nummularia* , would be beneficial for a more comprehensive understanding (Almalki and Alzahrani 2018; Khadivi 2023).

Looking ahead, under the optimistic scenario SSP1‐2.6, for the periods of 2041–2070 and 2071–2100, the habitat suitability for *Z. nummularia* is projected to undergo significant changes. Specifically, the species is projected to lose habitat suitability in Bushehr, Kohgiluyeh, and Boyer‐Ahmad Provinces, with a significant contraction expected in western Fars. Conversely, under this scenario, its potential distribution is predicted to shift towards higher latitudes in Khuzestan, with a new potential habitat emerging in Ilam Province. The pessimistic SSP5‐8.5 scenario for 2041–2070 predicts an expansion of the potential distribution of *Z. nummularia* in Khuzestan and Ilam Provinces, moving further north. However, projections for 2071–2100 indicate a significant reduction in suitable habitats, raising the risk of extinction of the species. Under this scenario, *Z. nummularia* habitats are expected to become unsuitable in Fars, Bushehr, Kohgiluyeh, and Boyer‐Ahmad, and southern Khuzestan, whereas new habitats may emerge in the higher latitudes of Ilam Province. Overall, despite these challenges, the predicted distribution of *Z. nummularia* is expected to exhibit an increasing trend in habitat suitability. Several studies, including Tarnian et al. (2021) and Khanal et al. (2022), support the observed patterns of change in the distribution of *Z. nummularia* .

In summary, future projections for *Z. spina‐christi* and *Z. nummularia* revealed a complex interplay between climate change and ecological resilience. Geographical barriers and the complex topography of the region, characterized by a hot and arid Saharo‐Sindian climate, along with the heavy weight of seeds (Zandiehvakili and Khadivi 2021), and competition from native and invasive species, such as *Prosopis juliflora* , may hinder *Z. spina‐christi* and *Z. nummularia* from adapting to suitable habitats in the future (Wakie et al. 2014). Given their limited seed dispersal abilities and specific physiological traits, such as drought tolerance thresholds, limited tolerance to temperature extremes, and slow growth rates, these species are particularly susceptible to climate change and lack the capacity to migrate to habitats predicted by species distribution models. As these species respond to the evolving landscape of their habitats, it is crucial to understand their responses to environmental changes to safeguard biodiversity and maintain ecosystem health in an unpredictable future.

### 4.2 Conservation Implications

Biodiversity within arid ecosystems is remarkable; however, these regions are highly vulnerable to environmental disturbances, including climate change and droughts. Unfortunately, the recovery process in such environments is notably slow, often resulting in irreversible harm and far‐reaching consequences for various components of these dry ecosystems (Wichmann et al. 2003; Rinawati et al. 2013; Isbell et al. 2015; Müller and Bahn 2022). The success of our efforts to monitor the natural habitats of two specific species, *Z. spina‐christi* and *Z. nummularia* , under both current and projected climate conditions depends on whether these species have the capacity and willingness to migrate to suitable habitat areas. Regrettably, these species appear ill equipped to adapt to the challenges posed by climate warming while maintaining their existing habitats. Consequently, their survival may depend on shifting their range to more suitable areas, which represents a species‐level adaptive response to changing environmental conditions. Although these species may face challenges in adapting to climate warming in their habitats, protecting their current populations remains crucial. These populations maintain genetic diversity, act as stepping stones for range shifts, and may persist in local microclimates that offer temporary refuge. A strategic conservation approach is vital to facilitate both survival and their role as biological refuge for other species. Over time, these species have contributed to the strengthening of biodiversity, enhanced ecosystem productivity, and increased resilience, essentially providing insurance for the ecosystem.

Additionally, considering both biotic and abiotic factors, and the multifaceted threats posed by climate change, habitat restoration efforts can focus on ecologically suitable areas identified through species distribution models (SDMs) and climate projection analyses. *Z. spina‐christi* and *Z. nummularia* are likely to be located in regions with favorable microclimatic conditions, such as the foothills of the Zagros Mountains and other semi‐arid transitional zones in southwestern Iran. By combining remote sensing techniques with field surveys, potential vacant niches can be mapped and prioritized for habitat restoration. Furthermore, the implementation of ex situ conservation (e.g., seed banks and controlled propagation) and assisted migration programs could enhance species resilience by establishing populations in newly suitable areas predicted under future climate scenarios.

### 4.3 Scope and Model Limitations

While the predictive models used in this study exhibited strong performance in delineating the climate niche of the studied species, we acknowledge certain limitations in our predictions. Although climatic variables are crucial in defining a species' ecological niche on a macro‐scale, they do not exclusively determine the ecological niche of a species (Searcy and Shaffer 2016).

This discrepancy arises from two key ecological considerations. First, there is an important distinction between a species' fundamental niche, the full range of environmental conditions under which it can survive, and its realized niche, which is constrained by factors such as biotic interactions, dispersal limitations, and geographical barriers (Soberón and Peterson 2005). In our case, limited seed dispersal capacity, competition with native and invasive species such as *Prosopis juliflora* , and the complex topography of the region may prevent *Z. spina‐christi* and *Z. nummularia* from occupying climatically suitable areas. Second, our species distribution models (SDMs) were based exclusively on climatic variables, without incorporating other environmental factors such as soil characteristics or species interactions. Climate is a dominant driver of species distributions at broad spatial scales, and relying solely on climatic predictors can oversimplify the ecological reality, potentially overestimating suitable habitats and overlooking constraints at finer scales (Araújo and Guisan 2006; Elith and Leathwick 2009).

Recognizing both the conceptual limitations of niche theory and the methodological constraints of climate‐only SDMs is essential for interpreting the results of our projections and understanding the full range of factors that shape species distribution under climate change. The integration of multiple data types, including climate, topography, soil properties, and remotely sensed data, can lead to more robust and biologically meaningful predictions (Parra et al. 2004; Bobrowski et al. 2018; Cobos and Peterson 2023).

Our predictive models primarily focused on climatic factors and excluded other biotic and abiotic factors. As a result, while these models effectively capture the influence of climate, they may not offer a comprehensive understanding of how other factors, such as species interactions, competition, and resource availability, can limit species distribution (Mod et al. 2015; Poggiato et al. 2025).

Anthropogenic activities significantly affect species distributions and introduce complexities that challenge predictive models. Land use change, overgrazing, deforestation, and urbanization are the key drivers of these impacts (Pei et al. 2024; Yates et al. 2010). These human‐induced changes can alter biodiversity patterns, ecosystem functioning, and species interactions in ways that are difficult to account for using traditional species distribution models (Razgour et al. 2020).

These approaches should also consider the multiple threats posed by anthropogenic activities and their interactions with climate change (Den Elzen and Schaeffer 2002; Yates et al. 2010; Razgour et al. 2020; Pei et al. 2024).

Thus, although our models highlight the importance of climate in determining species distribution, integrating other ecological factors is essential for a more holistic assessment of potential changes in the range of species due to climate change.

Other elements, such as land use and drought alterations, should also be viewed as interrelated variables warranting investigation in subsequent research endeavors because they are influenced by global warming (De Kort et al. 2020; Zangiabadi et al. 2021). Some studies have found that incorporating both dynamic (climate) and static (topography and soil) variables can improve the accuracy of species distribution models and provide more realistic predictions under climate change scenarios (Zangiabadi et al. 2021). Future research should focus on developing integrated approaches that consider these multiple factors to better predict and mitigate the impact of global change on biodiversity (Leroux et al. 2013; De Kort et al. 2020; Habel et al. 2021; Zangiabadi et al. 2021).

Given the species' relationship to their climatic niche and the challenging conditions typical of the Khalijo‐Omanian hot and arid ecosystem, the proposal is to implement a comprehensive strategy and immediate conservation measures aimed at safeguarding these two invaluable species (Arshad et al. 2024). However, to validate the capacity of these species to adapt to suitable habitats, it is vital to consider a range of factors, including biological interactions, topographic variations, and edaphic conditions, across different geographical scales. This approach can significantly enhance the accuracy of predictions (Turner et al. 2007; Williams and Newbold 2020).

## 5 Conclusions

This study provides critical insights into the potential distribution of *Z. spina‐christi* and *Z. nummularia* under current and future climate scenarios in the arid Khalijo‐Omanian ecosystem. Our findings indicate that both species face significant challenges in maintaining their existing habitats owing to climate change, with future projections suggesting a shift towards more suitable regions, particularly in areas with specific microclimatic conditions. The combined modeling approaches employed offer a comprehensive understanding of their potential responses, emphasizing that ecological traits, such as thermal tolerance and dispersal abilities, are critical in shaping the resilience of these species to climate change. By identifying ecologically suitable areas through species distribution modeling, this study offers a framework for prioritizing habitat protection and restoration. Given the ecological role of these species as biological refuge for other organisms, safeguarding their populations is essential for maintaining biodiversity and ecosystem stability in the region. Implementing targeted conservation measures, such as establishing ecological corridors and facilitating assisted migration, restoring habitat connectivity, and mitigating anthropogenic pressures, can enhance the long‐term survival of these species. Future research should expand these findings by incorporating additional environmental factors (e.g., biological interactions, land‐use changes, and edaphic conditions) and refining predictive models across diverse geographic scales. This integrated approach will improve our capacity to anticipate species responses to climate change and inform more effective conservation plans.

## Author Contributions

**Saeed Behzadi:** formal analysis (equal), investigation (equal), writing – original draft (equal). **Gholamabbas Ghanbarian:** conceptualization (equal), data curation (equal), funding acquisition (equal), project administration (equal), writing – review and editing (equal). **Rasool Khosravi:** conceptualization (equal), formal analysis (equal), funding acquisition (supporting), methodology (equal), software (equal). **Roja Safaeian:** investigation (supporting), resources (equal), writing – review and editing (equal). **Hamid Reza Pourghasemi:** software (supporting), validation (supporting), writing – review and editing (equal).

## Conflicts of Interest

The authors declare no conflicts of interest.

## Supporting information


Table S1

## Data Availability

Data are available from the Dryad Digital Repository: https://doi.org/10.5061/dryad.j6q5745w.
