# Supplementary material for: Habitat Suitability of Ziziphus spina‐christi and Ziziphus nummularia in a Changing Climate in the Khalijo‐Omanian Zone, Iran
Source: Ecol Evol. 2025 May 26;15(5):e71406. doi: 10.1002/ece3.71406 (PMC12105918; doi:10.1002/ece3.71406)
Supplement: Supplementary file 1 — Table S1 [file ECE3-15-e71406-s001.docx]

**Supplementary Material**

**Table S1 The ODMAP Protocol**

| **Section** | **Subsection** | **Element** | **Value** |
| --- | --- | --- | --- |
| Overview | Authorship | Study title | Navigating Tomorrow: Predicting Habitat Suitability for Two *Ziziphus* Species in a Changing Climate |
|  |  | Author names | Saeed Behzadi; Gholamabbas Ghanbarian; Rasool Khosravi; Roja Safaeian; Hamid Reza Pourghasemi |
|  |  | Contact | g.ghanbarian@gmail.com; ghanbarian@shirazu.ac.ir |
|  | Model objective | Model objective | Mapping, forecast, and niche analysis |
|  |  | Target output | Changes in spatial patterns of two Ziziphus species in response to climate warming |
|  | Focal Taxon | Focal Taxon | Ziziphus spina-christi and Ziziphus nummularia |
|  | Location | Location | Southern Iran |
|  | Scale of Analysis | Spatial extent | 44.03346, 63.32512, 25.05846, 39.77512 (xmin, xmax, ymin, ymax) |
|  |  | Spatial resolution | ~ 1 km (30") |
|  |  | Temporal extent | 1981-2010; 2041-2070 & 2071-2100 |
|  |  | Temporal resolution | 30 years |
|  |  | Boundary | Political |
|  | Biodiversity data | Observation type | Field survey |
|  |  | Response data type | Presence/pseudo-absence |
|  | Predictors | Predictor types | Climatic |
|  | Hypotheses | Hypotheses | How will the spatial patterns of the two Ziziphus species change in response to climate warming? |
|  | Assumptions | Model assumptions | Ensemble species distribution models |
|  | Algorithms | Modelling techniques | GLM; GBM & MaxEnt |
|  |  | Model ensemble | Mean |
|  | Software | Software | R version 4.2.3 and biomod2 version 4.2-5-2 package |
| Data | Biodiversity data | Ecological level | Species |
|  |  | Cleaning | To reduce spatial autocorrelation and prevent model overfitting, presence data were spatially filtered to a minimum distance of 1-km from each other using the SDM toolbox. |
|  | Data partitioning | Training data | 80% sub-sampling (split sample) |
|  |  | Testing data | 20% sub-sampling (split sample) |
|  | Predictor variables | Predictor variables | 19 bioclimatic variables |
|  |  | Data sources | CHELSA ver. 2.1 (<http://chelsa-climate.org>) |
|  |  | Spatial extent | 45.65819, 63.32486, 25.05819, 34.14153(xmin, xmax, ymin, ymax) |
|  |  | Spatial resolution | The spatial resolution of bioclimatic variables is 30" (_~_1-km). |
|  |  | Coordinate reference system | WGS1984 |
|  |  | Temporal extent | 1981-2010 |
|  |  | Temporal resolution | 30 years |
|  | Transfer data | Data sources | The future climate variables were obtained from the 6^th^ assessment report of the Intergovernmental Panel on Climate Change (IPCC AR6), were extracted from the CHELSA ver. 2.1. |
|  |  | Spatial extent | 45.65819, 63.32486, 25.05819, 34.14153(xmin, xmax, ymin, ymax) |
|  |  | Spatial resolution | ~ 1 km (30") |
|  |  | Temporal extent | 2041-2070 & 2071-2100 |
|  |  | Temporal resolution | 30 years |
|  |  | Models and scenarios | Models: GFDL-ESM4  Scenarios: SSP1-2.6 & SSP5-8.5. |
| Model | Multicollinearity | Multicollinearity | Hierarchical cluster analysis with Pearson correlation coefficient (\|r\|<0.7) |
|  | Model settings | Model settings (fitting) | For all three modeling algorithms, the default parameters provided by the biomod2 R package. |
| Assessment | Performance statistics | Model evaluation with independent test data | Boyce Index |
| Prediction | Prediction output | Prediction unit | Probability of presence (predicting ensemble models) |
